# Characterization of a periplasmic nitrate reductase in complex with its biosynthetic chaperone

**DOI:** 10.1111/febs.12592

**Published:** 2013-12-09

**Authors:** Jennifer M. Dow, Sabine Grahl, Richard Ward, Rachael Evans, Olwyn Byron, David G. Norman, Tracy Palmer, Frank Sargent

**Affiliations:** ^1^ Division of Molecular MicrobiologyCollege of Life SciencesUniversity of DundeeUK; ^2^ CRUK Nucleic Acid Structure Research GroupCollege of Life SciencesUniversity of DundeeUK; ^3^ School of Life SciencesUniversity of GlasgowUK; ^4^ Department Biologie I, Botanik, BiozentrumLudwig‐Maximilians‐Universität MünchenGroßhadernerStrasse 2‐4Planegg‐MartinsriedGermany

**Keywords:** chaperone, periplasmic nitrate reductase, protein–protein interaction, Tat pathway, twin‐arginine signal peptide

## Abstract

*Escherichia coli* is a Gram‐negative bacterium that can use nitrate during anaerobic respiration. The catalytic subunit of the periplasmic nitrate reductase NapA contains two types of redox cofactor and is exported across the cytoplasmic membrane by the twin‐arginine protein transport pathway. NapD is a small cytoplasmic protein that is essential for the activity of the periplasmic nitrate reductase and binds tightly to the twin‐arginine signal peptide of NapA. Here we show, using spin labelling and EPR, that the isolated twin‐arginine signal peptide of NapA is structured in its unbound form and undergoes a small but significant conformational change upon interaction with NapD. In addition, a complex comprising the full‐length NapA protein and NapD could be isolated by engineering an affinity tag onto NapD only. Analytical ultracentrifugation demonstrated that the two proteins in the NapDA complex were present in a 1 : 1 molar ratio, and small angle X‐ray scattering analysis of the complex indicated that NapA was at least partially folded when bound by its NapD partner. A NapDA complex could not be isolated in the absence of the NapA Tat signal peptide. Taken together, this work indicates that the NapD chaperone binds primarily at the NapA signal peptide in this system and points towards a role for NapD in the insertion of the molybdenum cofactor.

**Structured digital abstract:**

- NapD and NapA bind by x ray scattering (View interaction)
- NapA and NapD physically interact by molecular sieving (View interaction)
- NapA and NapD bind by electron paramagnetic resonance (View interaction)

**Abbreviations**
AUC: analytical ultracentrifugation
HiPIP: high potential iron protein
IMAC: immobilized metal ion affinity chromatography
MoCo: Mo‐*bis*‐molybdopterin guanine dinucleotide cofactor
MTSL: *S*‐(2,2,5,5‐tetramethyl‐2,5‐dihydro‐1H‐pyrrol‐3‐yl)methylmethanesulfonothioate
PELDOR: pulsed electron–electron double resonance
SAXS: small‐angle X‐ray scattering
SDSL: site‐directed spin labelling
SE: sedimentation equilibrium
SEC: size exclusion chromatography
SV: sedimentation velocity
Tat: twin‐arginine translocation

## Introduction

The facultative anaerobe and model bacterium *Escherichia coli* is capable of considerable respiratory flexibility. In the absence of oxygen it is able to use a range of terminal electron acceptors, the most energetically favourable of which is nitrate. To maximize the availability of nitrate from the environment, *E. coli* produces three nitrate reductase enzymes 1. All three of these enzymes contain molybdenum at their active sites, which is bound to an inorganic cofactor as Mo‐*bis*‐molybdopterin guanine dinucleotide (MoCo). Nitrate reductase‐A is the major nitrate reductase produced under anaerobic growth conditions in the presence of nitrate 4, while nitrate reductase‐Z is structurally related to nitrate reductase‐A but is produced at low levels until entry into stationary phase 5. Both nitrate reductase‐A and nitrate reductase‐Z are located at the cytoplasmic face of the inner membrane 6.

**Figure 1:**
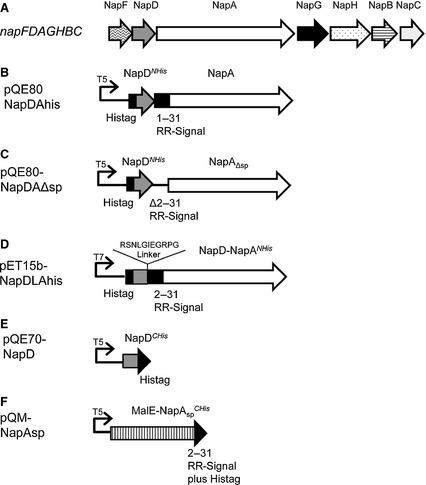
The *Escherichia coli nap* operon and constructs used in this study to investigate NapA–NapD interactions. (A) A cartoon representing the structure of the napFDAGHBC operon located at 46.5 min on the *E. coli* chromosome. The names of the protein products of the genes are given above the arrows. (B) An overexpression vector based on pQE80 (Qiagen) encoding full‐length NapA and NapD with an N‐terminal hexa His‐tag (NapD^*NHis*^). The natural transcriptional and translational coupling between napD and napA is maintained. (C) A pQE80‐based expression vector encoding NapD^*NHis*^ and NapA lacking its entire Tat signal peptide comprising Lys2‐Ala31 (NapA_Δsp_). (D) A pET15b‐based expression vector (Novagen/Merck, Darmstadt, Germany) encoding a fusion between NapD^*NHis*^ and NapA. The amino acid sequence of the linker is shown. (E) An overexpression vector based on pQE70 (Qiagen) encoding a C‐terminally hexa His‐tagged NapD (NapD^*CHis*^). (F) An overexpression vector based on pQE60 (Qiagen) encoding the mature sequence of maltose binding protein (MalE), fused to the NapA signal peptide via a polyasparagine linker and a factor Xa recognition sequence, and a C‐terminal hexa His‐tag.

The third *E. coli* nitrate reductase, Nap, is located in the periplasmic compartment. The catalytic subunit NapA is a 90 kDa protein that binds a [4Fe‐4S] cluster in addition to the molybdenum cofactor 7. NapA is encoded, along with its periplasmic di‐heme *c*‐type cytochrome redox partner NapB, in the seven gene *nap* operon (Fig. 1A). NapA is exported to the periplasm in a folded form by the twin‐arginine protein transport (Tat) pathway 10, which is a translocation system dedicated to the export of fully folded proteins. In *E. coli* approximately two‐thirds of the 28 known Tat substrates bind one or more redox cofactors 12.

Proteins are targeted to the Tat pathway by N‐terminal tripartite signal peptides comprising polar n‐ and c‐regions that flank a hydrophobic h‐region. Tat‐targeting signal peptides contain a conserved S‐R‐R‐X‐F‐L‐K consensus amino acid motif where the consecutive arginine residues are almost invariant 13. This motif is specifically recognized by the membrane‐bound TatC component 15 and the signal peptide is usually cleaved off at a late stage of protein transport by a periplasmically facing signal peptidase 16.

Some twin‐arginine signal peptides have dual functions. In addition to their Tat‐targeting role, they also act as binding sites for biosynthetic chaperones 17. A well‐studied example of a signal‐peptide‐binding biosynthetic chaperone is TorD, which binds to the twin‐arginine signal peptide of its cognate Tat substrate trimethylamine‐*N*‐oxide reductase, TorA, and at a second site close to the mature N‐terminus 18. TorD binding facilitates molybdenum cofactor insertion into TorA by maintaining the apoprotein in an open conformation, simultaneously shielding the substrate from interacting with the Tat machinery while allowing the cofactor to insert. This process has been termed ‘Tat proofreading’ 17 since it ensures that immature substrates are not exported to the periplasm in haste. The binding of the molybdenum cofactor to TorA induces folding of the enzyme that necessitates translocation via the Tat pathway (reviewed in 17). Indeed, it is thought that all Tat‐dependent molybdoenzymes receive their cofactors in the cytoplasm prior to export 17.

NapA is subject to Tat proofreading prior to export by the Tat pathway. NapD is a small (9.3 kDa) cytoplasmic protein that is essential for Nap activity. It has been shown to bind with a nanomolar dissociation constant to the isolated NapA Tat signal peptide 22. Site‐directed mutagenesis has indicated that NapD recognizes an epitope that straddles the n‐ and h‐regions of the NapA signal peptide, including Arg6 and Lys10 that form part of the twin‐arginine consensus motif (Fig. 1A) 23. However, the precise role of NapD in the assembly of NapA remains unclear. To shed light on this process, in this work we have isolated and characterized a complex of the two proteins. Our results indicate that the proteins are present in 1 : 1 stoichiometry and that the NapA precursor has a substantial degree of folding whilst in complex with NapD. In the absence of the NapA twin‐arginine signal peptide a stable complex could not be isolated, confirming that this is the major NapD‐binding epitope.

## Results

### The NapA signal peptide undergoes conformational rearrangement upon interaction with NapD

Previous biophysical studies on the isolated Tat signal peptides of SufI or high potential iron protein (HiPIP) have revealed that they are both largely unstructured in aqueous solution 24. It has recently been shown by NMR techniques that the NapA signal peptide adopts an α‐helical conformation when bound to NapD 23; however, it is not clear whether the helical form of the signal peptide is induced upon binding with NapD or whether it is an intrinsic feature of the alanine‐rich signal peptide. To investigate this, site‐directed spin labelling (SDSL) of the signal peptide and pulsed electron–electron double resonance (PELDOR) spectroscopy were carried out 26.

**Figure 2:**
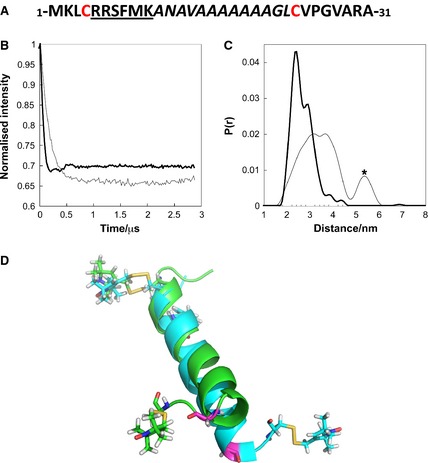
PELDOR analysis of the MTSL‐labelled MalE‐NapA_SP_ fusion protein in complex with NapD^*CHis*^. (A) Primary sequence of the NapA signal peptide with the Cys residues introduced at positions 4 and 24 that were the sites for spin label attachment marked in red. The background corrected dipolar evolution (B) and the Tikhonov‐derived distance distribution (C) for MalE‐NapA_SP_ alone (grey) or in complex with NapD (black). The asterisk in (C) indicates a long‐distance Tikhonov‐derived background removal artifact. (D) Comparison of bound, NMR‐derived NapA_SP_ helix (green; adapted from PDB code 2PQ4) versus free, generated helix (cyan). The positions of the spin labels in the two conformations of the signal peptide are also shown.

The NapA signal peptide can be produced in isolation when genetically fused to maltose binding protein (MalE) 22. Here, this system was modified such that serine residues at positions 4 and 24 of the NapA signal peptide were substituted for cysteine (Figs 2A and S1). Following purification, the resultant MalE‐NapA_SP_ S4/24C chimera was site‐specifically labelled with *S*‐2,2,5,5‐tetramethyl‐2,5‐dihydro‐1H‐pyrrol‐3‐yl)methylmethanesulfonothioate (MTSL), a thiol‐specific labelling reagent that contains a nitroxide radical. Note that MalE contains no endogenous cysteine residues. Labelling of the protein was confirmed by MALDI‐TOF mass spectrometry, with the labelled protein showing a mass shift from 47 500 Da to 47 834 Da, equivalent to the addition of two 186 Da MTSL groups (data not shown).

Preliminary experiments where MTSL‐labelled MalE‐NapA_SP_ S4/24C was mixed with C‐terminally His‐tagged, but otherwise native, NapD resulted in a loss of some of the spin labels from the NapA signal peptide. This behaviour was considered to be possibly due to the surface‐exposed native cysteine residues of NapD. The NapD cysteine residues (C8 and C32) are not conserved and a cysteine‐free variant of NapD was found to complement a Δ*napD* strain for restoration of NapA activity (data not shown). A NapD C8S C32A variant was therefore prepared and incubated with the MTSL‐labelled MalE‐NapA_SP_ S4/24C. In this case, the spin labels remained attached to the NapA signal peptide.

Next, the free MTSL‐labelled MalE‐NapA_SP_ S4/24C chimera, mixed at an equimolar ratio with the Cys‐less variant of NapD^*CHis*^, was examined using the PELDOR/DEER experiment 27 in order to measure the electron–electron dipolar coupling and deduce the distance distribution between spin labels. When the labelled MalE‐NapA_SP_ S4/24C fusion was examined alone, the distance distribution of the spin labels measured by electron paramagnetic resonance spectroscopy was narrower and shorter than that predicted by molecular dynamic simulations of an unstructured NapA peptide, indicating that the free signal peptide has adopted some degree of secondary structure (Figs 2B, S2 and S4). However, in the presence of NapD, the distance between the spin labels on MalE‐NapA_SP_ clearly changed (Fig. 2B,C; compare the MalE‐NapA_SP_ broad distance distribution centred at around 3.5 nm with the much narrower MalE‐NapA_SP_‐NapD complex distance distribution, main distance at 2.4 nm, minor distance at 2.9 nm). The EPR‐derived distance distribution of the complex is in good agreement with a calculated spin label distribution (Fig. S3) based on the NMR structure of the NapA signal peptide–NapD complex (PDB code 2PQ4). These data strongly suggest that NapD induces a conformational change in the NapA signal peptide upon binding.

The EPR data demonstrate that the NapA signal peptide alone is more structured than random coil, as shown by comparison with molecular dynamics simulation, and undergoes a structural alteration upon binding NapD. Furthermore, molecular dynamics simulations, in conjunction with the spin label distance distributions observed by EPR, suggest (Fig. S4B,C and associated text) that in the absence of NapD the NapA peptide is probably largely helical but may exhibit a conformational heterogeneity at Gly22. In the NapD bound form, Gly22 adopts a left‐handed helical conformation whereas in the unbound state this position may partially adopt a right‐handed helical conformation thus extending the length of the helix (Fig. 2D).

### Isolation of a stable NapDA complex

Although NapD can clearly interact tightly with the isolated NapA signal peptide, little is known about its interaction with the entire full‐length NapA precursor protein. To this end, the two proteins were co‐overproduced from a pQE80 expression vector. The cloning strategy maintained the natural translational coupling between the *napDA* genes whilst supplying the NapD protein with an N‐terminal hexa‐histidine affinity tag, NapD^*NHis*^ (Fig. 1B).

**Figure 3:**
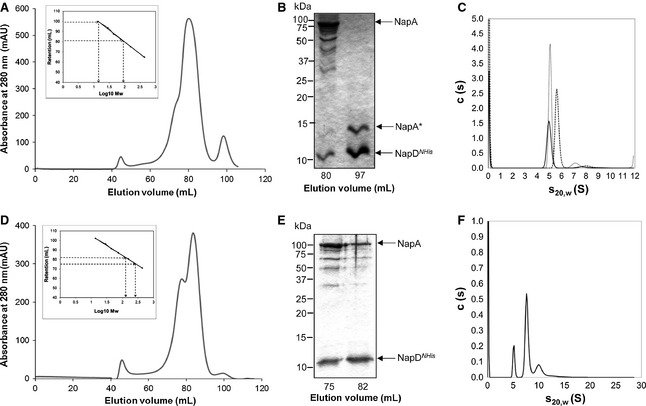
Isolation of a NapD^*NHis*^/NapA complex. (A), (D) NapD^*NHis*^‐ and NapA‐containing fractions after metal chelate chromatography that eluted with either 145 mm imidazole (A) or 235 mm imidazole (D) were pooled, concentrated and applied to a HiLoad 16/60 Superdex 200 Prep Grade size exclusion column. Eluted protein was monitored by measuring absorbance at 280 nm. The column was calibrated with the standard proteins ribonuclease (14 kDa), carbonic anhydrase (29 kDa), ovalbumin (44 kDa), conalbumin (75 kDa), aldolase (158 kDa) and ferritin (440 kDa), and the linear regression analysis is shown as inset boxes where (A) *R*^2^ = 0.9976, *y *= −23.50*x* + 126.83 and (D) *R*^2^ = 0.9988, *y *= −20.81*x* + 125.98. Mw, molecular mass. (B), (E) SDS/PAGE analysis (15% gels) of the indicated fractions from size exclusion chromatography shown in (A) and (D), respectively. The identities of NapD^*Nhis*^ and NapA were confirmed by tryptic digest mass spectrometry. NapA* indicates a mixture of degradation products of NapA. (C), (F) Analysis of the NapD^*Nhis*^/NapA complexes from (A) and (D), respectively. The *c*(*s*) distributions are derived via sedfit from SV data for varying concentrations of NapD^*Nhis*^/NapA: (C) 0.5 mg·mL^−1^ (solid line), 0.75 mg·mL^−1^ (dashed line), 1 mg·mL^−1^ (dotted line); (F) 0.5 mg·mL^−1^.

The soluble cell extract containing overproduced NapD^*NHis*^ and NapA was loaded onto a 5 mL Ni^2+^‐charged HisTrap‐HP immobilized metal ion affinity chromatography (IMAC) column and bound protein was eluted with a stepped gradient of imidazole. Four distinct peaks eluted from the column, of which the latter two (eluting at 145 and 235 mm imidazole, respectively) contained proteins of the predicted sizes of NapD^*NHis*^ and NapA (Fig. S5). The two NapDA‐containing protein fractions were separately pooled, concentrated and subjected to further purification by size exclusion chromatography (SEC) using a Hiload 16/60 Superdex 200 column. As shown in Fig. 3A, the protein sample that eluted from the IMAC column at 145 mm imidazole resolved into two peaks after SEC. The major peak eluted at a volume corresponding to a molecular weight of 98 kDa (Fig. 3A, inset) and was broad with a shoulder on the leading edge. SDS/PAGE analysis (Fig. 3B) revealed that it contained proteins corresponding to the known molecular weights of NapA and NapD^*NHis*^, and the identity of these proteins was confirmed by tryptic peptide mass fingerprint analysis. The major peak also contained a number of other proteins that cross‐reacted with a NapA antiserum which could be NapA degradation products (data not shown). The smaller peak, which eluted at a retention time corresponding to a molecular weight of 24 kDa, also contained two proteins. One of these was confirmed by tryptic peptide mass fingerprinting to be NapD^*NHis*^, and the other contained several peptides from both the N‐ and C‐terminal portions of the NapA polypeptide and would therefore appear to be a mixture of small NapA fragments. It can be concluded from these data that a stable complex of NapDA can be isolated but that NapA is prone to degradation during purification.

The second NapDA‐containing IMAC fraction, eluting at 235 mm imidazole (Fig. S5), partially resolved into two overlapping peaks following SEC, with retention times corresponding to masses of approximately 214 and 107 kDa (Fig. 3D). SDS/PAGE of these fractions showed that each contained major bands corresponding to the expected masses of NapA and NapD^*NHis*^ (Fig. 3E), which were subsequently confirmed by tryptic peptide mass fingerprinting. It was considered that the large peak could probably represent a dimeric form of the NapDA species found in the smaller peak. This hypothesis would also explain why this protein fraction eluted from the IMAC at a higher concentration of imidazole, since the presence of more than one NapD^*NHis*^ in the complex would result in delayed elution from the nickel affinity matrix.

### NapA and NapD^*NHis*^ are present at a 1 : 1 ratio in the NapDA complex

**Figure 4:**
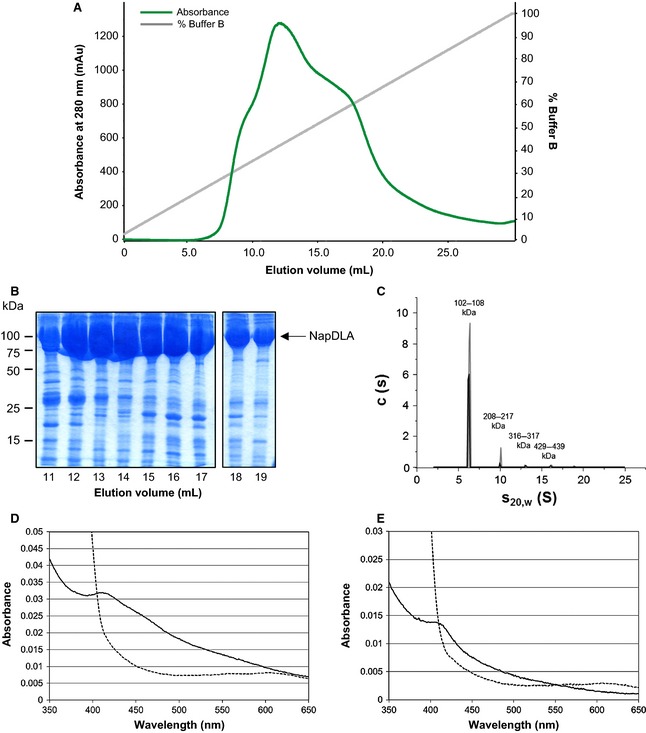
Evidence for the presence of a putative iron–sulfur cluster in the NapD^*Nhis*^/NapA complex and a NapDA fusion protein. (A) Crude cell extract containing an overproduced His‐tagged NapDA fusion protein (NapDLA) was loaded onto a His‐trap column. After elution of non‐specifically bound proteins by washing with buffer, an imidazole gradient of 0–500 mm was applied to the column. Elution of protein was monitored by measuring absorbance at 280 nm. (B) Laemmli sample buffer was added to 10 μL of each of the indicated fractions in a 1 : 1 ratio and the samples were separated by SDS/PAGE (10% acrylamide). (C) The oligomeric state of the NapDLA protein was determined by SV AUC at protein concentrations of 0.25 mg·mL^−1^ (black line) and 0.5 mg·mL^−1^ (grey line). (D), (E) Spectroscopic analysis of IMAC‐purified NapD^*Nhis*^/NapA and NapDLA samples. Immediately after IMAC purification pooled NapD^*Nhis*^/NapA (D) or NapDLA (E) were diluted 1 : 100 in buffer A and air‐oxidized (solid line) followed by dithionite‐reduced (dotted line) absorption spectra were recorded.

To determine the stoichiometry of NapA and NapD^*NHis*^ in the isolated complexes, sedimentation velocity (SV) and sedimentation equilibrium (SE) analytical ultracentrifugation (AUC) was undertaken. SV AUC showed that the NapA and NapD^*NHis*^ complex eluting from the SEC column with an estimated mass of around 100 kDa was present predominantly as a single species in solution, with an *s* value in the range 5–6. A minor peak was also detected between 7 and 8 S. There is no evidence of dissociation of the components during the experiment (Fig. 3C). The infinite dilution sedimentation coefficient (*s*^0^_20,w_) of NapD^*NHis*^/NapA was determined to be 5.37 S. By contrast, the approximately 200 kDa NapD^*NHis*^/NapA complex sample contained two main species, a large species with an apparent sedimentation coefficient of 7.6 S and a smaller species of 5.2 S, indicating dissociation of the 200 kDa complex into a species with a sedimentation coefficient comparable to the 100 kDa NapD^*NHis*^/NapA complex (Fig. 3F). The most likely explanation for this is that the larger complex is a dimeric form of the smaller NapD^*NHis*^/NapA complex. To corroborate this analysis, an artificial fusion protein was constructed where the C‐terminus of NapD was genetically fused through an RSNLGIEGRPG linker sequence to the extreme N‐terminus of the NapA Tat signal peptide (Fig. 1D). The N‐terminus of NapD was supplied with a hexa‐histidine tag to facilitate purification. The crude cell extract containing the overproduced fusion protein was applied to an IMAC column and the fusion protein was observed to elute as a broad peak (Fig. 4A,B). To estimate the solution molecular mass of this fusion protein, SV AUC was undertaken (Fig. 4C). The majority of the protein in the sample had an approximate molecular mass of 102–108 kDa, which correlates well with the predicted molecular mass of the NapDLA fusion polypeptide (106 278 Da).

Next, SE AUC was used to accurately determine the molecular mass of the NapD^*NHis*^/NapA complex. Data were collected for the approximately 100 kDa NapD*NHis*/NapA complex (fraction 80 in Fig. 3B) at three different wavelengths (260, 280 and 300 nm) and speeds (18 000, 12 000 and 49 000 r.p.m., data not shown). The experimentally determined molecular mass for this NapD^*NHis*^/NapA complex was 99.7 kDa, which is slightly smaller than the predicted size for a 1 : 1 complex of the component proteins (103.3 kDa). These data, however, point to the two proteins in the NapD^*NHis*^/NapA complex being present in a 1 : 1 ratio.

### Absorption spectroscopy indicates that the NapDA complex may contain iron

During purification of the NapD^*NHis*^/NapA complex and NapDLA fusion protein it was noted that the NapDA‐containing fractions were deep brown in colour following IMAC. This brown colour was gradually lost if the sample was stored at 4 °C or during further purification steps. Scanning absorption spectroscopy was undertaken on the NapD^*NHis*^/NapA and NapDLA proteins following IMAC and spectra with a broad absorption peak at ~ 420 nm were observed, which can be indicative of the presence of an [Fe‐S] cluster (Fig. 4D,E) 29. This spectral feature was lost when the samples were reduced by the addition of excess sodium dithionite (Fig. 4D,E). Attempts to collect EPR spectra of the reduced samples were unsuccessful (data not shown).

To determine the molybdenum content of both protein samples they were analysed by inductively coupled plasma mass spectrometry. Comparisons of the ratios between the amount of protein complex analysed and the amount of molybdenum detected indicated that only 1% of the protein had molybdenum present (not shown).

### NapA produced without its signal peptide does not stably interact with NapD

To ascertain whether NapD is able to interact with NapA in the absence of the NapA twin‐arginine signal peptide, a construct was designed where N‐terminally His‐tagged NapD could be co‐overproduced with NapA lacking amino acids 2–31 of its signal peptide (hereafter termed NapA_Δsp_; Fig. 1C).

**Figure 5:**
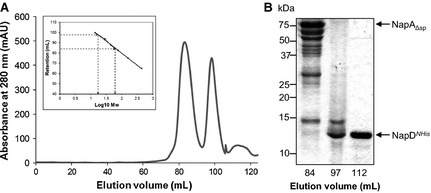
An unstable complex is formed between NapD and NapA in the absence of the NapA signal peptide. (A) NapD^*Nhis*^‐ and NapA_Δsp_‐containing fractions after metal chelate chromatography were pooled, concentrated and applied to a HiLoad 16/60 Superdex 200 Prep Grade size exclusion column. Eluted protein was monitored by measuring absorbance at 280 nm. The column was calibrated with the standard proteins ribonuclease (14 kDa), carbonic anhydrase (29 kDa), ovalbumin (44 kDa), conalbumin (75 kDa), aldolase (158 kDa) and ferritin (440 kDa), and the linear regression analysis is shown as an inset. *R*^2^ = 0.9988, *y *= −20.81*x* + 125.98. Mw, molecular mass. (B) SDS/PAGE analysis (15% gel) of the indicated fractions from size exclusion chromatography shown in (A). The identities of NapD^*Nhis*^ and NapA_Δsp_ were confirmed by tryptic digest mass spectrometry.

**Figure 6:**
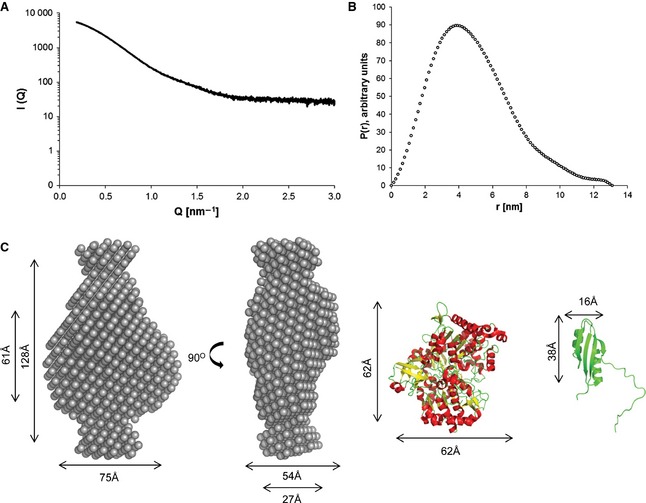
SAXS characterization of the NapD^*N*^^*his*^/NapA complex. (A) SAXS scattering curve for the NapD^*N*^^*his*^/NapA complex. *Q* is the scattering vector and *I* represents intensity. (B) Distance distributions *P*(*r*) for NapD^*N*^^*his*^/NapA. (C) Two different views of the *ab initio* model of NapD^*N*^^*his*^/NapA, generated using dammin 55.The X‐ray structure of *Escherichia coli *NapA (PDB code 2NYA 9) and the NMR structure of *E. coli *NapD (PDB code 2JSX 22) are shown to scale on the right.

The soluble extract of aerobically grown *E. coli* cells overproducing NapD^*NHis*^ and NapA_Δsp_ was applied to an IMAC column. After elution of bound proteins with an imidazole gradient, NapD^*NHis*^‐containing fractions were identified by SDS/PAGE. Some NapA_Δsp_ protein was found to co‐elute with NapD^*NHis*^ from the column (not shown) and to analyse this complex further the NapD^*NHis*^/NapA_Δsp_‐containing fractions were pooled, concentrated and applied to a SEC column. As shown in Fig. 6A, SEC analysis of this sample resulted in the elution of three protein peaks at retention times corresponding to approximately 116, 22 kDa and a peak outside the calibrated range of the column. SDS/PAGE of these fractions showed the presence of NapA_Δsp_ exclusively in the first peak; however, this fraction contained no evidence of the NapD^*NHis*^ protein (Fig. 5B). Instead NapD^*NHis*^ was found in the later fractions, indicating that, in the absence of the NapA signal peptide, the complex was not interacting strongly enough to survive the chromatography step. We conclude that NapD^*NHis*^ has some affinity for the NapA_Δsp_ protein but that the NapA signal peptide contains the primary epitope for NapD binding.

### Small angle X‐ray scattering analysis of the NapD^*NHis*^/NapA complex

To assess the overall shape of the purified NapD^*NHis*^/NapA complex, a low resolution envelope of the complex was generated using small angle X‐ray scattering (SAXS). The SAXS scattering and distance distribution curves are shown in Fig. 6A,B, respectively, and the parameters of the complex extracted from these features are given in Table 1. The complex has a maximum length of 130 Å and an estimated molecular mass of 103 kDa from the Porod volume, which is in good agreement with the molecular weight of the complex deduced from AUC. The simulated annealing approach was subsequently used to create *ab initio* models for the complex. Multiple low resolution models were generated for NapD^*NHis*^/NapA using the program dammif 30. These models were then clustered, averaged and filtered to create a single representative model, which is shown in Fig. 6C. The model shows a central density flanked by two distal lobes, which would be consistent with the components of the complex having a substantial degree of folding. This assertion is supported by analysis of the Kratky plot which indicates that both components are folded (not shown).

**Table 1:** SAXS parameters obtained from the scattering data and GNOM modelling for the NapD^*NHis*^/NapA complex.

*R*_g_ (Guinier)	*R*_g_ *p* (*r*)	*D*_max_ *p* (*r*)	Porod volume	MW from Porod
36.7 Å	36.6 Å	130 Å	164.0 nm^3^	103 kDa

## Discussion

In this study the complex formed between the Tat substrate protein NapA and its biosynthetic chaperone NapD has been characterized. Previous work had shown that there is a high affinity binding site for NapD on the NapA signal peptide 22. Here SDSL followed by PELDOR analysis of the NapA signal peptide has been used to show that the NapA signal peptide undergoes some structural changes upon NapD binding. However, in the absence of NapD, the signal peptide still shows significant structure that is probably largely helical, which contrasts with other twin‐arginine signal peptides that appear to be largely unstructured in aqueous solution 24. The NapA signal peptide is unusual because it has a string of seven consecutive alanine residues within the h‐region, and such alanine repeats are known to stabilize helical structure 31. It is interesting to note that the N‐terminal region of the catalytic subunit of nitrate reductase‐A which is a ‘remnant’ twin‐arginine signal peptide and a binding site for the NarJ chaperone 32, also shows a helical conformation when analysed in solution 33.

A stable complex of the NapA precursor and an N‐terminally hexa‐histidine tagged NapD can be isolated. The stability of the complex can be largely ascribed to the interaction of NapD with the signal peptide portion of the precursor, since in the absence of the signal peptide the complex is destabilized. This is supported by the findings of Chan *et al*. 34 who showed that there was no detectable interaction between NapD and the mature sequence of NapA in the bacterial two‐hybrid system. Several approaches, including AUC and SAXS, indicated that the two proteins were present in the NapDA complex in a 1 : 1 stoichiometry. There was some tendency for the complex to dimerize, but the hetero‐tetrameric form was not stable and dissociated during AUC or SEC.

The NapDA complex was brown in colour, and absorption spectroscopy indicated that an [Fe‐S] cluster might be present. However, metal analysis indicated that the complex was largely devoid of molybdenum. The lack of molybdenum was not surprising given that the NapDA complexes studied here were produced in aerobically grown cells and that biosynthesis of the molybdenum cofactor is greatly induced only under anaerobic conditions 35. Moreover, it has been shown previously that for the catalytic subunits of the diimethylsulfoxide reductase DmsA and the nitrate reductase‐A NarG the MoCo is inserted subsequent to [4Fe‐4S] assembly, and that oxidative damage of this cluster to a [3Fe‐4S] blocks insertion of MoCo 35. The results presented here corroborate these previous findings and, given the stable attachment of NapD to NapA observed here, suggest that NapD is additionally required for NapA maturation at a stage after [Fe‐S] cluster assembly into the enzyme.

The SAXS analysis of the NapDA complex suggests that the NapA precursor has a high degree of folding (data not shown). However, the SAXS‐derived envelope is significantly larger than the sum of the fully folded NapD and NapA structures. This strongly suggests that NapA is present in a more ‘open’ conformation in the complex, which would be consistent with a lack of MoCo in the NapA precursor. The shape of the complex is not dissimilar in overall architecture to the SAXS‐derived model for the TorAD complex 21. However, unlike the TorAD complex where the component high‐resolution structures of TorA and TorD could be well fitted into the SAXS envelope by rigid body modelling, the rigid body models created for the NapDA SAXS data had less volume than the corresponding *ab initio* models, even when integrating flexibility into the model by allowing flexibility in the orientation of domain IV of NapA (which is the only domain of Nap/DMSO reductase family enzymes that is formed from a contiguous stretch of polypeptide chain and which is known to be flexible in the TorA precursor 21). This indicates that the folding present in the crystal structure is not an accurate reflection of the folding of NapA in the complex, i.e. all four domains of NapA are likely to be partially unfolded in the complex (Fig. S6).

Previous analysis of the precursor of the molybdoenzyme TorA in complex with the TorD chaperone has shown that the C‐terminal 173 amino acids of TorA, comprising the entirety of domain IV, are sensitive to trypsin digestion, indicating flexibility in this part of the protein 21. Since this forms a lid over the MoCo binding site, it has been speculated that closing of the domain IV lid is the final step in the maturation of TorA and may promote release of the bound TorD chaperone 21. Limited trypsinolysis of the NapDA complex indicated that the analogous domain IV region of NapA is also accessible to trypsin (data not shown), consistent with the MoCo binding site being open and empty. However, unlike TorA, the NapA precursor was sensitive to trypsin digestion at several sites within the N‐terminal 315 amino acids (not shown), supporting the conclusion from the SAXS analysis that the NapA precursor is much less compact than mature NapA.

Finally, it should be considered that additional chaperone proteins may be required to complete the maturation of NapA. It is known that some complex Tat substrate proteins such as the small subunits of periplasmically facing hydrogenases require more than one chaperone for their assembly 37. In this context, a second cytoplasmic accessory protein encoded by the *nap* operon NapF has also been shown to interact with NapA 39. NapF was not overproduced in any of the experiments described here and is likely to have been present at extremely low levels since the *nap* operon is repressed under aerobic growth conditions 3. In future it would be interesting to ascertain the role of NapF in the NapA proofreading process.

## Materials and methods

### Plasmid and strain construction

To overproduce N‐terminally His‐tagged NapD (NapD^*NHis*^) along with untagged NapA, the encoding genes were amplified using oligonucleotides NapD‐pQE80start (5′‐GCGCGGATCCCACATAACTGGCAAGTTTGCAGC‐3′) and NapA‐pQE80stop (5′‐GCGCAAGCTTTTACACCTTCTCCAGTTTGACCGC‐3′) with *E. coli* strain MC4100 41 chromosomal DNA as template. The resultant PCR product was digested with *Bam*HI and *Hind*III and cloned into similarly digested pQE80 (Qiagen, Hilden, Germany)) to give pQENapDA. This plasmid was subsequently used as a template to produce a construct encoding NapD and NapA lacking its N‐terminal signal peptide. To amplify *napD*, oligonucleotides pQE forward (5′‐CCCGAAAAGTGCCACCTG‐3′) and NapAdelsig1 (5′‐GCGCCCATGGTGTTTCCTCACCTTGCTCTTCC‐3′) were used. The resultant PCR product was digested with *Bam*HI and *Nco*I. To amplify the truncated *napA*, oligonucleotides NapAdelsig2 (5′‐GCGCTCATGATTGTTGGTCAGCAGGAAGCC‐3′) and NapAb4Cla (5′‐CGCTTTAAACAGCTTGGACG‐3′) were used. The resultant PCR product was digested with *Rca*I and *Cla*I. pQE80 NapDA was digested with *Bam*HI and *Cla*I and the two amplified and digested PCR products were cloned into the digested pQE80 NapDA in a three‐way ligation. The resulting construct was designated pQE80 NapDAΔsp.

To construct a fusion between NapD and NapA with an RSNLGIEGRPG amino acid linker sequence, a synthetic DNA sequence was designed. This encompassed the whole of the *napD* sequence except the stop codon, and part of *napF*, the linker coding sequence and the first 500 base pairs of *napA*. The synthetic DNA was supplied cloned between the *Kpn*I and *Bam*HI sites of pBluescript SK(+). This construct was subsequently sub‐cloned between the *Kpn*I and *Bam*HI sites of plasmid pMAK705 42 and the resulting plasmid was used to move the *napDA* fusion allele onto the chromosome of *E. coli* strain LCB2048 (*nar25*(*narGH*), *thi*‐*1*,* leu*‐*6*,* thr*‐*1*,* rpsL175*,* lacY*, Km^R^, *narZ*::Ω, Spec^R^ 43) to give strain SGDLA10. Subsequently, DNA covering the entire *napDA* fusion allele lacking the *napA* start codon was amplified with oligonucleotides NapDLA forward (5′‐CGCGCCTCGAGCACACTAACTGGCAAGTTTGC‐3′) and NapDLA reverse (5′‐CGCGCGGATCCTTACACCTTCTCCAGTTTCAG‐3′) and SGDLA10 chromosomal DNA as template. The resultant PCR product was digested with *Xho*I and *Bam*HI and cloned into similarly digested pET15b to give plasmid pET15b‐NapDLAhis.

The QuikChange™ (Stratagene, La Jolla, CA, USA) site‐directed mutagenesis procedure with oligonucleotide pair 5′‐CCCGGGATGAAACTCTGTCGTCGTAGCTTTATG‐3′ and 5′‐CATAAAGCTACGACGACAGAGTTTCATCCCGGG‐3′ was used to introduce a Ser to Cys codon substitution at position 4 of the NapA signal peptide present on the MalE‐NapAsp fusion protein encoded on plasmid pQM‐NapA. Subseqently a Ser to Cys substitution was also introduced at codon 24 of the NapA signal peptide using oligonucleotides 5′‐GCGGCTGCCGGTCTCTGCGTGCCGGGCGTTGCC‐3′ and 5′‐GGCAACGCCCGGCACGCAGAGACCGGCAGCCGC‐3′ to give pQM‐NapASSCC. To substitute the native cysteine residues in NapD encoded on the pQE70‐NapD construct, primer pairs 5′‐ACTAACTGGCAAGTTAGCAGCCTGGTCGTGCAG‐3′ and 5′‐CTGCACGACCAGGCTGCTAACTTGCCAGTTAGT‐3′ were used to introduce a C8S substitution and 5′‐AACGCCTTTCCCGGCGCTGAAGTTGCTGTCAGC‐3′ and 5′‐GCTGACAGCAACTTCAGCGCCGGGAAAGGCGTT‐3′ to introduce a C32A substitution. All constructs were fully sequenced to ensure that no mismatches had been introduced inadvertently during the cloning procedure.

### Preparation of recombinant proteins

NapD^*NHis*^/NapA proteins encoded on plasmids pQE80‐NapDAhis, pQE80‐NapDAΔsp and pET15b‐NapDLAhis were overproduced and purified similarly. A single colony of freshly transformed *E. coli* BL21(DE3) [F^−^*ompT hsdS*(r_B_^−^ m_B_^−^) *gal dcm* λ(DE3)] was used to inoculate a 5 mL pre‐culture in LB medium supplemented with 100 μg·mL^−1^ ampicillin. After 6 h, the entire pre‐culture was used to inoculate a 500 mL culture, which was grown aerobically at 37 °C until an *A*_600_ of 0.6 was reached. Production of plasmid‐encoded proteins were induced by addition of 1 mm isopropyl β‐d‐thiogalactopyranoside, followed by a temperature shift to 18 °C. After 16 h cells were harvested and resuspended (10 mL per 1 g cells) in buffer A (50 mm Tris/HCl, 200 mm KCl, 1 mm dithiothreitol, 25 mm imidazole, pH 7.5). Protease inhibitor (EDTA‐free complete protease inhibitor cocktail, Roche), lysozyme and DNase were subsequently added and cells were lysed using an Emulsiflex C3 high pressure homogenizer. Cell debris was removed by a short centrifugation step (15 min at 18 850 ***g***) followed by removal of membranes (200 000 ***g*** for 1 h).

The supernatant obtained following ultracentrifugation was filtered through a 0.22 μm membrane filter (Millipore/Merck, Darmstadt, Germany) before loading onto a 5 mL His‐Trap column (GE Healthcare Life Sciences, Little Chalfont, Buckinghamshire, UK) equilibrated with buffer A. After extensive washing with buffer A, hexa‐histidine tagged proteins were subsequently eluted using a gradient of 25–500 mm imidazole (note that buffer B in Fig. 4 and Fig. S5 is buffer A containing 500 mm imidazole). NapD^*NHis*^/NapA‐containing fractions were identified by SDS/PAGE, pooled and concentrated to 2 mL using a Vivaspin 20 spin concentrator (10 kDa cutoff; Sartorius, Epsom, Surrey, UK). The concentrated samples were then individually loaded onto a calibrated Hiload 16/60 Superdex 200 Prep grade (GE Healthcare) gel filtration column equilibrated with buffer C (50 mm Tris/HCl, 200 mm KCl, 1 mm dithiothreitol, pH 7.5). NapD^*NHis*^/NapA‐containing fractions were again identified by SDS/PAGE.

NapD^*CHis*^, encoded by plasmid pQE70‐NapD, was overproduced and purified as described previously 22. The Cys‐substituted variant of the ϕMalE‐NapAsp^*His*^ fusion protein was purified by IMAC under denaturing conditions and subsequently refolded as described previously 23.

Protein concentration was determined by the method of Lowry 44. For absorbance spectroscopy, dithionite‐reduced and air‐oxidized absorbance spectra were recorded between 350 and 650 nm wavelength using a Lambda UV/Vis spectrophotometer (Perkin Elmer, Waltham, MA, USA).

### Metal analysis

Metal content was analysed by inductively coupled plasma atomic emission spectrometry/inductively coupled plasma mass spectrometry and was provided as a service by the School of Chemistry at the University of Edinburgh.

### Site‐directed spin labelling of MalE‐NapA_SP_

Purified and refolded MalE‐NapA_SP_ S4/24C was reduced with 20 mm dithiothreitol followed by immediate buffer exchange with seven volumes of 20 mm Tris/HCl, pH 7.6, 50 mm NaCl using a Vivaspin 20 column (10 000 molecular weight cutoff). The protein was labelled with a 10‐fold molar excess of MTSL (Toronto Research Chemicals, Toronto, Ontario, Canada). Labelling was allowed to proceed for 4 h at room temperature followed by an overnight incubation at 4 °C. Unbound MTSL was removed by a final buffer exchange to deuterated 20 mm Tris/HCl, pH 7.6, 50 mm NaCl. Labelling was confirmed by mass spectrometry (FingerPrints Proteomics Facility, University of Dundee). One hundred micromoles MalE‐NapA_SP_ S4/24C was mixed in a 1 : 1 ratio with purified NapD^*CHis*^ in the presence of 50% D8‐glycerol (Cambridge Isotope Laboratories) and stored at −80 °C until EPR measurements were carried out.

### PELDOR analysis

PELDOR experiments were executed using a Bruker ELEXSYS E580 spectrometer (Bruker, Billerica, MA, USA) operating at X‐band with a dielectric ring resonator and a Bruker 400 U second microwave source unit. All measurements were made at 50 K with an overcoupled resonator giving a *Q* factor of approximately 100. The video bandwidth was set to 20 MHz. The four pulse, dead‐time free, PELDOR sequence was used, with the pump pulse frequency positioned at the centre of the nitroxide spectrum; the frequency of the observer pulses was increased by 80 MHz. The observer sequence used a 32 ns π‐pulse; the pump π‐pulse was typically 16 ns. The experiment repetition time was 4 ms, and the number of scans was sufficient to obtain a suitable signal with 50 shots at each time point.

For data analysis, briefly the experimentally obtained time domain trace was processed to remove any unwanted intermolecular couplings, which is called the background decay. Tikhonov regularization was then used to simulate time trace data that give rise to distance distributions *P*(*r*) of different peak widths depending on the regularization factor α. The α term used was judged by reference to a calculated L curve. The L curve is a plot of the α term against quality of fit, measured by mean square deviation between the experimental data and simulation. The most appropriate α term to be used is at the inflection of the L curve, since this provides the best compromise between smoothness (artifact suppression) and fit to the experimental data. PELDOR data were analysed using the deer analysis 2006 software package 45. The dipolar coupling evolution data were corrected for background echo decay using a homogeneous three‐dimensional spin distribution. The starting time for the background fit was optimized to give the best fit Pake pattern in the Fourier transformed data and the lowest root mean square deviation background fit. The Pake pattern can allow distance determination using the equation

$$f_{\mathrm{Dip}}\left(r,\theta \right)=\frac{\mu_{B}^{2}g_{A}g_{B}\mu_{0}}{2\pi h}.\frac{1}{r_{\mathrm{AB}}^{3}}\left(3\cos^{2}\theta -1\right)$$

where θ is the angle between the spin–spin vector *r* and the direction of the applied magnetic field, μ_B_ is the Bohr magneton, μ_0_ is the permeability of free space, *g*_A_ and *g*_B_ are the *g* values for the two spin labels A and B, and *r* is the spin–spin distance, assuming the exchange coupling constant can be neglected. If a resolved perpendicular turning point feature is observed in the spectrum a mean distance can be inferred.

For spin label dynamics, coordinates were taken from PDB codes 2PQ4 and mutated within pymol 46 to replace required amino acid positions with cysteine. Parameter and topology files for MTSSL were created using prodg 47. Coordinates for the MTSSL spin label were generated and minimized using the program ghemical 48 and then melded with the protein structures by common atom superposition within pymol. Molecular dynamics, using xplor‐nih 49, were carried out on the whole complex, NapA alone and a pymol generated helix, in order to generate distributions for each spin label attached to a cysteine mutant site. Backbone C, N and O atoms were restrained by harmonic function to initial positions and the unrestrained atoms were allowed to move under molecular dynamics at a temperature of either 300 K for the whole complex or 400 K for NapA or the synthetic helix alone. Structures were taken at regular intervals and the distance between pairs of spin label nitroxide nitrogen atoms were calculated and binned into 1 Å groups to generate synthetic distance distributions.

In the molecular visualization package pymol, each of the 20 NMR model structures was taken and the NapA peptide mutated at the positions 4 and 24 for cysteines. MTSL was then added on at these positions. All 20 structures were then used in a simplistic molecular dynamics, repel only and no water.

### Analytical ultracentrifugation

SV and SE experiments were conducted at 4 °C using a Beckman Optima XL‐I analytical ultracentrifuge and an An‐50 Ti eight hole rotor (Beckman Coulter, Brea, CA, USA). For SV, samples (360 μL) at concentrations from 0.5 to 1.0 mg·mL^−1^ along with buffer (50 mm Tris/HCl, pH 7.5, 200 mm KCl, 1 mm dithiothreitol and 10% (v/v) glycerol) as reference solvent were loaded into 12 mm path‐length charcoal‐filled epon‐double‐sector centrepieces and spun at 49 000 r.p.m., and a series of 130 scans was collected using interference and absorbance optics (280 nm). Data were recorded every 7 min over a radial range of 5.80–7.25 cm, and a radial step size of 0.005 cm was used in the case of absorbance optics. For interference optics the laser delay was adjusted prior to the run to obtain high‐quality interference fringes. The partial specific volumes ($\overset{¯}{\upsilon }$) of NapA and NapD were calculated from their amino acid compositions using the program sednterp 50 which was also used to calculate the density and viscosity of the buffer at 4 and 20 °C. SV data were analysed using the program sedfit 51. Sedimentation boundaries were modelled as numerical finite element solutions of the Lamm equation using *c*(*s*) analysis. The apparent sedimentation coefficients were then corrected to standard conditions of temperature and solvent to obtain *s*_20,w_.

SE data were acquired at two speeds (18 000 and 12 000 r.p.m.) using interference optics and absorbance optics at three different wavelengths (260, 280 and 300 nm). Scans were recorded at 3 hourly intervals until thermodynamic equilibrium was confirmed [using the programme winmatch (Jeffrey Lary, University of Connecticut, Storrs, CT, USA)] for samples (90 μL) at concentrations from 0.5 to 1.0 mg·mL^−1^. Data were recorded over a radial range of 6.8–7.25 cm, with the laser delay adjusted before the run. Data were analysed using the program sedphat 52.

### Small angle X‐ray scattering (SAXS)

SAXS data using purified NapD^*NHis*^/NapA were recorded at the P12 BioSAXS beamline at the PETRA III Deutsches Elektronen‐Synchrotron (DESY, Hamburg, Germany). Sample–detector distance was 3.1 m for an X‐ray wavelength of 0.124 nm. 25 μL protein and buffer samples were loaded in a flow‐through quartz capillary cell at 7 °C. Sample volume exposed to the beam was approximately 10 μL. Data (20 0.5 s frames) were assessed for radiation damage.

The two‐dimensional diffraction patterns were normalized to an absolute scale and azimuthally averaged to obtain intensity profiles at the beamline. Solvent contributions (buffer backgrounds collected before and after every protein sample) were averaged and subtracted from the associated protein sample using the program primus 53 and the data were analysed using programs from the atsas package 54.

## Supplementary Material

**Fig. S1.** The positions of the spin labels introduced into the NapD signal peptide.
**Fig. S2.** PELDOR data of spin labelled MalE‐NapA_SP_ (S4R1, S24R1) in the absence and presence of NapD.
**Fig. S3.** Comparison of the Tikhonov‐derived distance distribution with a synthetic distance distribution generated by molecular dynamics simulations on each of the 20 NMR models of the NapA/NapD complex.
**Fig. S4.** The Tikhonov‐derived distance distribution for unbound MalE‐NapA_SP_ compared with various dynamic simulations.
**Fig. S5.** Immobilized metal affinity chromatography of NapD^*NHis*^.
**Fig. S6.** Rigid body modelling of the NapDA complex.
