# Supplementary material for: Characterization of a periplasmic nitrate reductase in complex with its biosynthetic chaperone
Source: FEBS J. 2013 Dec 9;281(1):246–60. doi: 10.1111/febs.12592 (PMC4159696; doi:10.1111/febs.12592)
Supplement: Supplementary file 1 — Fig. S1. The positions of the spin labels introduced into the NapD signal peptide. Fig. S2. PELDOR data of spin labelled MalE‐NapASP (S4R1, S24R1) in the absence and presence of NapD. Fig. S3. Comparison of the Tikhonov‐derived distance distribution with a synthetic distance distribution generated by molecular dynamics simulations on each of the 20 NMR models of the NapA/NapD complex. Fig. S4. The Tikhonov‐derived distance distribution for unbound MalE‐NapASP compared with various dynamic simulations. Fig. S5. Immobilized metal affinity chromatography of NapDNHis. Fig. S6. Rigid body modelling of the NapDA complex. [file febs-281-246-s1.zip › febs12592-sup-0001-FigS1-S6.pdf]

the FEBS Journal
Wiley Blackwell
www.febsjournal.org

# Characterization of a periplasmic nitrate reductase in complex with its biosynthetic chaperone

Jennifer M. Dow, Sabine Grahl, Richard Ward, Rachael Evans,
Olwyn Byron, David G. Norman, Tracy Palmer and Frank Sargent

DOI: 10.1111/febs.12592

# Characterisation of a periplasmic nitrate reductase in complex with its biosynthetic chaperone

Jennifer M. Dow, Sabine Grahl, Richard Ward, Rachael Evans, Olwyn Byron, David G. Norman, Tracy Palmer and Frank Sargent

# SUPPLEMENTARY INFORMATION

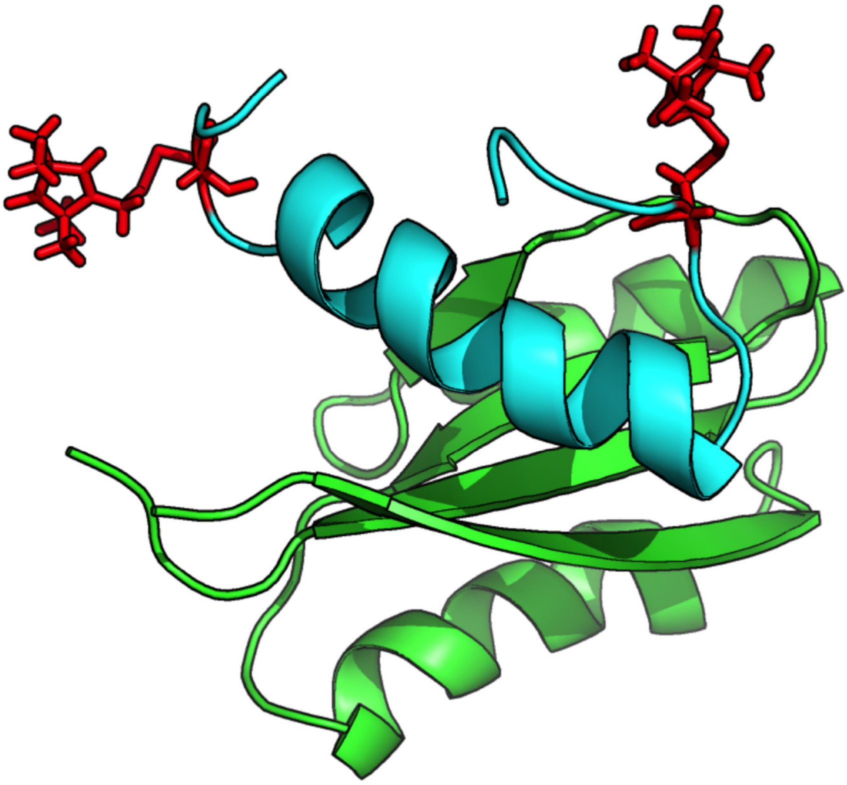

**Figure S1. The positions of the spin labels introduced into the NapD signal peptide.** The solution structure of NapD in complex with NapA MalE-NapA$_{SP}$ (amino acids 1-35) is shown, with the spin labels attached to NapA MalE-NapA$_{SP}$ and colored red (adapted from PDB code 2PQ4).

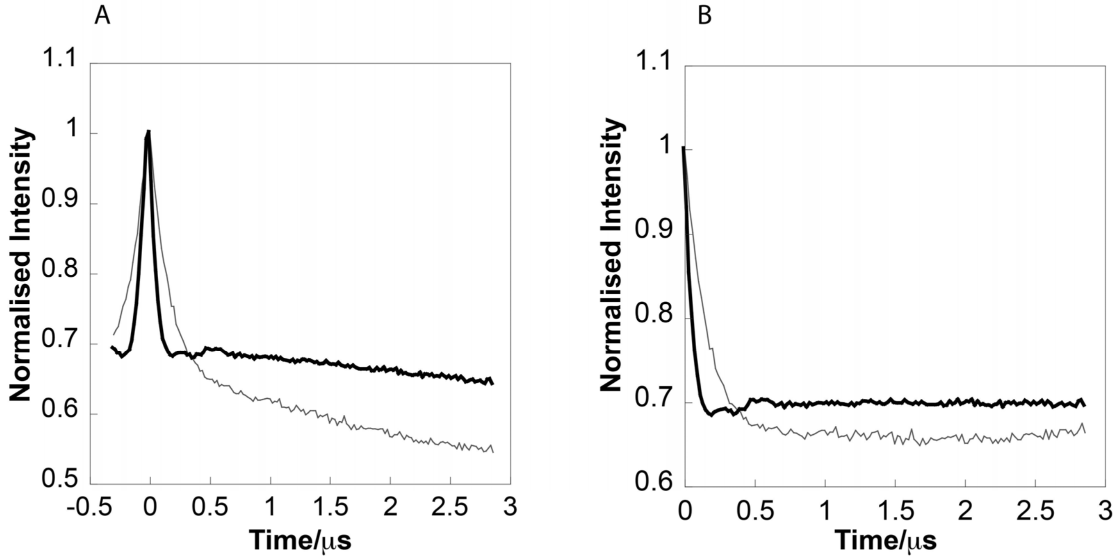


**Figure S2. PELDOR data of spin labeled MalE-NapA$_{SP}$ (S4R1, S24R1) in absence and presence of NapD. (A.)** PELDOR experimental data. **(B.)** Background corrected experimental data. Data for MalE-NapA$_{SP}$ alone are represented by the thin grey line whilst data for the MalE-NapA$_{SP}$-NapD complex are represented by the thick black line.

Note that the PELDOR data from MalE-NapA$_{SP}$ showed a decrease in the distance between spin labels upon binding to NapD as shown by the initial slope of the PELDOR data as illustrated in Fig S2A uncorrected, and S2B background corrected. The data for the NapD-bound form exhibited oscillations in the signal indicating a relatively defined and uniform structure. The data for the unbound form of MalE-NapA$_{SP}$ did not show oscillations in the signal after the initial drop. The data for the unbound form is indicative of some greater degree of structural heterogeneity.

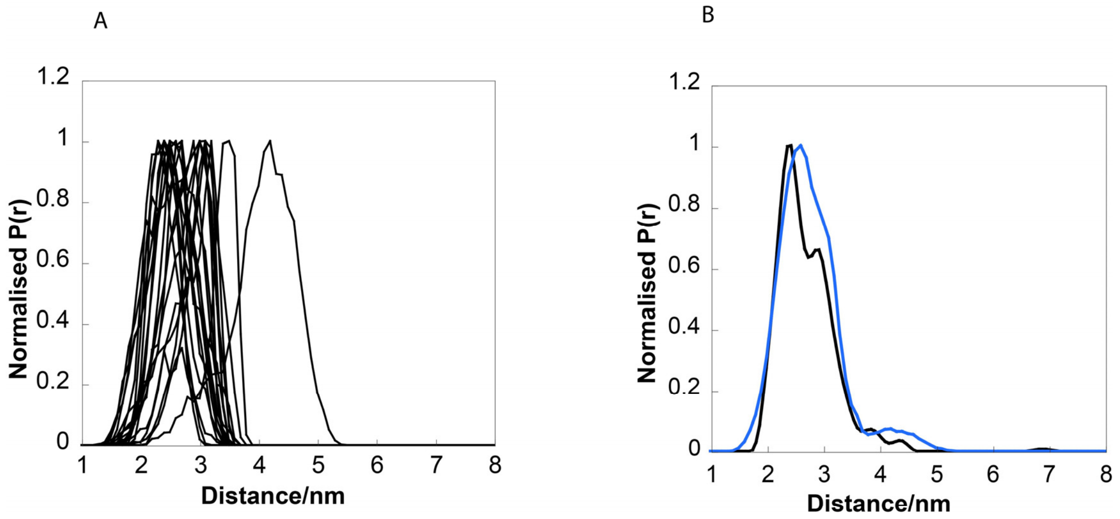


**Figure S3**. **Comparison of the Tikhonov-derived distance distribution with a synthetic distance distribution generated by molecular dynamic simulations on each of the twenty NMR models of the NapA-NapD complex.**

**(A.)** All twenty normalised synthetic distance distributions from the NapAsp-NapD data in PDB code 2PQ4. **(B.)** Normalised average of the twenty synthetic distance distributions (blue line), PELDOR distribution from MalE-NapA$_{SP}$-NapD complex (black line).

Note that the family of twenty NMR structures of the NapA$_{SP}$-NapD complex was used to generate twenty synthetic distance distributions between spin labels (Fig S3A), using the molecular dynamics software package Xplor-NIH. The average of the twenty distance distributions was then calculated, normalised, and compared with the Tikhonov derived distance distribution (Fig S3B). The high degree of similarity between the experimentally determined and computer generated distance distributions in both main distance (2.6 cf. 2.4 nm) and distribution width, strongly suggests that the spin labeled peptide is in the same conformation as the reported NMR structure.

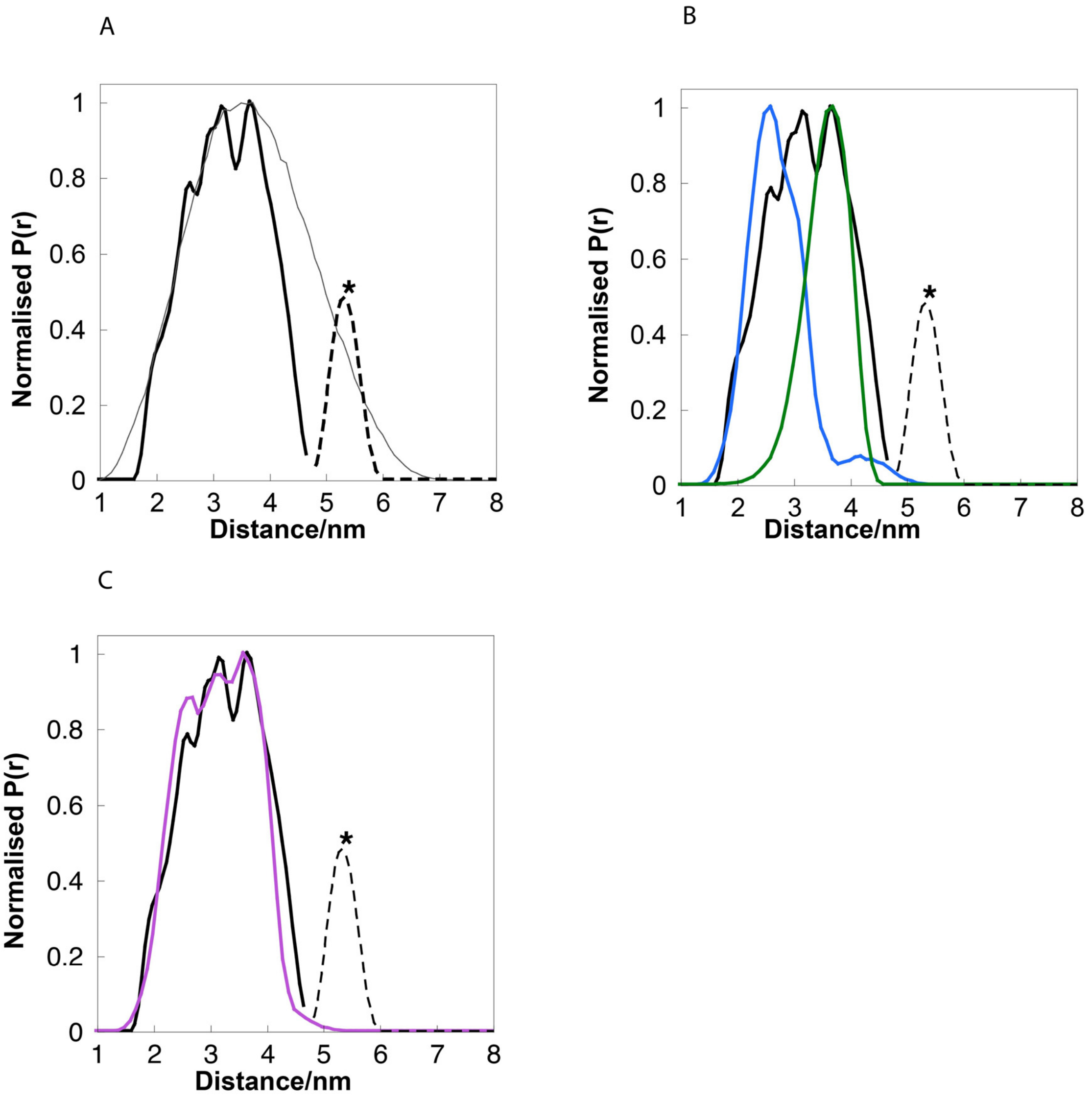


**Figure S4**. **The Tikhonov-derived distance distribution for unbound MalE-NapA$_{SP}$ compared to various dynamic simulations.** The Tikhonov-derived distance distribution for unbound MalE-NapA$_{SP}$ (thick black line, baseline removal artifact indicated by dotted line and *) compared to various dynamic simulations. **(A.)** Unrestrained simulation of random coil NapA$_{SP}$ (thin grey line). **(B.)** Average distribution derived from NapA$_{SP}$ NMR models (blue line), distance distribution derived from simulation of NapA$_{SP}$ built as a regular alpha-helix (green line). **(C.)** Mixture of NapA$_{SP}$ derived from NMR models and regular alpha helix (purple line).

Note that unrestrained molecular dynamics simulations of the spin labeled $NapA_{SP}$ gave a synthetic spin label distance distribution that might be expected from a completely random coil peptide (Fig S4A). Comparison to the EPR-derived distance distribution for MalE-$NapA_{SP}$ (Fig S4A) indicated that the unbound $NapA_{SP}$ was more structured that random coil. Simulations of $spNapA_{SP}$ in which the main structures were restrained to either the conformation seen in the NMR structure or restrained to a perfect alpha helical form, were compared to the EPR-derived distance distribution (Fig S4B). An almost equal combination of the two simulated distance distributions shown in Fig S4B resulted in the distribution shown in Fig S4C that bears a remarkable similarity to the experimentally determined distribution of the free MalE-$NapA_{SP}$. The simplest interpretation of the data is that in free solution $NapA_{SP}$ exists as an equilibrium between the form shown bound to NapD in the NMR structure and a form in which glycine 22 of the peptide adopts a right handed helical conformation. The central helical character of $NapA_{SP}$ appears to be conserved in both the free and bound state.

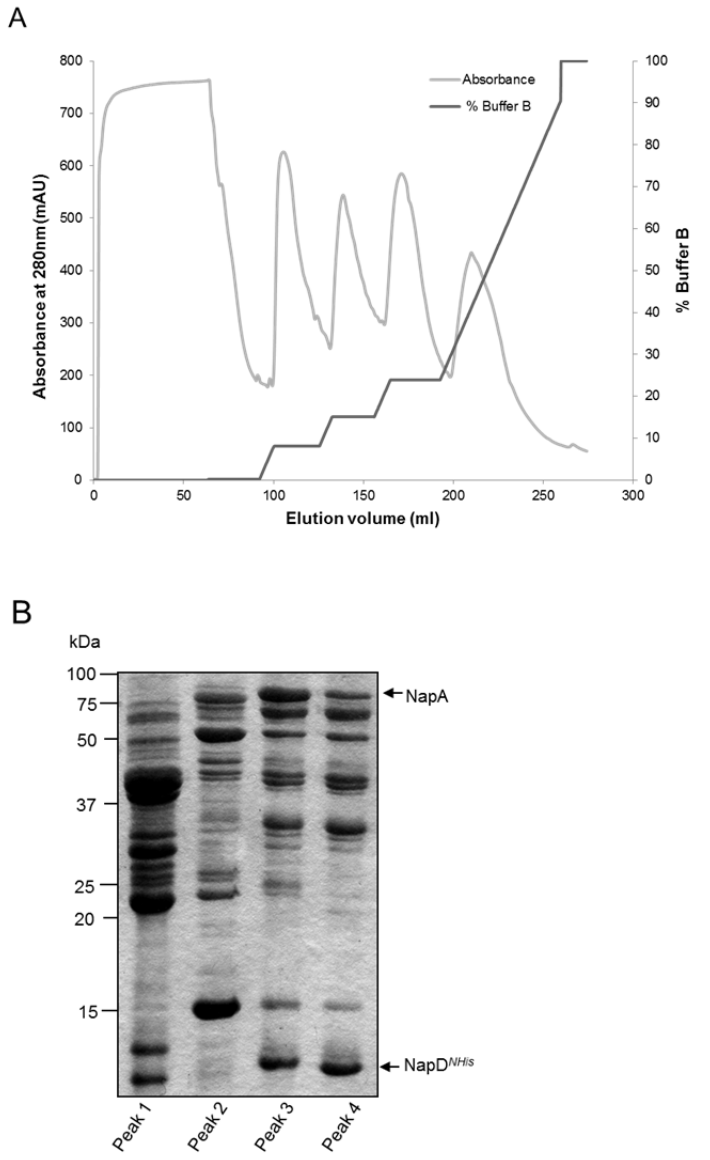


**Figure S5. Immobilised metal affinity chromatography of NapD$^{NHis}$. (A)** Crude cell extract containing overproduced NapD$^{NHis}$ and NapA was loaded onto a His-trap column. After elution of non-specifically bound proteins by washing with buffer, an imidazole gradient of 25-500 mM was applied to the column. Elution of protein was monitored by measuring absorbance at 280nm. **(B)** SDS-PAGE analysis (15 % acrylamide) of the non-concentrated fractions from each of the four peaks eluting over the imidazole gradient. The identities of NapD$^{NHis}$ and NapA are indicated.

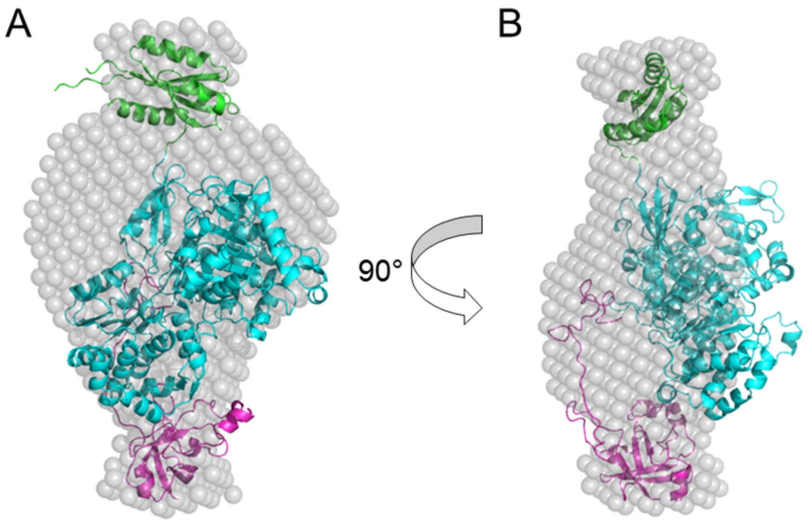


**Figure S6**. **Rigid body modelling of the NapDA complex.** Rigid body modelling, using SASREF [1], was conducted using the component parts of the complex; the modelled structure of *E. coli* NapD in complex with the NapA signal peptide [2] and *E. coli* NapA [3], which was split into two parts domains I-III (amino acids 2-630; shown in cyan) and domain IV (amino acids 631-791; shown in magenta). Constraints were applied to ensure the final residue of the signal peptide of NapA and the first residue of the mature NapA were within 5 Å of each other. **(B)** is vertcially rotated 90° in respect to **(A)**.

[1] Petoukhov MV & Svergun DI (2005) Global rigid body modeling of macromolecular complexes against small-angle scattering data *Biophys J* **89,** 1237-1250.

[2] Grahl S, Maillard J, Spronk CA, Vuister GW & Sargent F (2012) Overlapping transport and chaperone-binding functions within a bacterial twin-arginine signal peptide *Mol Microbiol* **83**, 1254-1267.

[3] Jepson BJ, Mohan S, Clarke TA, Gates AJ, Cole JA, Butler CS, Butt JN, Hemmings AM & Richardson DJ (2007) Spectropotentiometric and structural analysis of the periplasmic nitrate reductase from *Escherichia coli J Biol Chem* **282**, 6425-6437.
